# *Pangasius* Fish Skin and Swim Bladder as Gelatin Sources for Hard Capsule Material

**DOI:** 10.1155/2021/6658002

**Published:** 2021-08-27

**Authors:** Mala Nurilmala, Noviyan Darmawan, Erin Apriliani Wulandari Putri, Agoes M. Jacoeb, Tun Tedja Irawadi

**Affiliations:** ^1^Department of Aquatic Products Technology, Bogor Agricultural University (IPB University), Bogor 16680, Indonesia; ^2^Halal Science Center, LPPM, Bogor Agricultural University (IPB University), Bogor 16680, Indonesia; ^3^Department of Chemistry, Bogor Agricultural University (IPB University), Bogor 16680, Indonesia

## Abstract

In this paper, we report the extraction and characterization of gelatin from the abundant industrial fishery waste of *Pangasius* skin and swim bladder and its application as the base material for hard capsule shells. The yield of gelatin ranged between 19 and 23%, content of moisture is 7.6–9.2%, ash is 1.1–1.7%, pH is 4.1–5.2, gel strength is 238–278 bloom, and viscosity is 65–74.7% mP. SDS-PAGE showed all gelatins have chains of *α*1, *α*2, and *β*-peptides. The skin, swim bladder, and mixed gelatins were successfully used in the production of hard capsule shells. The dimensions, weight, disintegration time, and water content properties of the hard capsules from these *Pangasius* wastes were akin to the standards of commercial capsules.

## 1. Introduction

Fish industries generate more than 50% of waste from the total weight of the processed fish [1]. Recently, to reduce the amount of waste generated, a lot of research studies have focused on turning these byproducts into a value-added product. Nam et al. [2] reported the conversion of catfish excesses into bioactive components such as fish protein hydrolysate (FPH), hydroxyapatite (HA), and lipid fraction. In addition, several studies have successfully reported the extraction of gelatin from the fish byproducts such as tuna skin [3, 4], *Pangasius* skin [5], and Chinese giant salamander [6].

Gelatin is an animal-based protein derived mostly from the bones and skins of cows and pigs, collected from the slaughterhouse. It has been used in the food and pharmaceutical industries due to its ability to form a hydrogel in the pH range, devoid of ionic assistance or other additives [7]. Moreover, the physical form of the gel solution changed between the gel and solution reversibly according to temperature changes. Hence, gelatin has been employed as a base material for hard capsules. Interestingly, more than 15% of marketed drugs are delivered in gelatin-based hard capsules [8]. Hard gelatin capsules are preferred because they are easier to swallow than tablets, mask the bitter taste, extend the drug shelf life, and allow facile drug identification by using various colors and patterns on the capsule surface.

However, lately, there are concerns to use extracted gelatin from bovine skins and bones due to the possibility of conveying the bovine spongiform encephalopathy (BSE) diseases and preferably lifestyles such as vegetarian. Several countries have abandoned the use of the porcine-based capsule due to religious causes. The alternative for these mammalian-based gelatins is gelatin extracted from fish processing wastes including the skin, bone, swim bladder, and scale. Nevertheless, the commercial application of fish gelatin has been impeded due to the lower gelling temperature and gel strength in comparison to mammalian-based gelatin, particularly, gelatin from cold water fish.

Stimulatingly, several reports have shown that tropical fish have physicochemical characteristics parallel to bovine and porcine gelatins. The extraction of gelatin from *Pangasius* skin had a yield of 14.94% with an acidic method and had a yield of 14.30% with the base method [5]. The extraction of gelatin from the catfish swim bladder had a yield of 13.5% with an acidic and base process [9]. Interestingly, there are no published reports of the application of this fish waste gelatin as the base material for hard capsule shells. Thus, in this paper, we used the abundant and cheap tropical fish waste of *Pangasius* skin and swim bladder for gelatin extraction and then applied as a material for hard capsule shells.

## 2. Materials and Methods

### 2.1. *Pangasius* Waste Materials and Preparation

The frozen *Pangasius* skin and swim bladder were obtained from the waste of the fish fillet industry located in West Java, Indonesia. The raw materials were cleaned from the meat, fat, and other impurities using a sharp knife and washed with running water. The cleaned product was cut into a square shape with a size of ±1 × 1 cm and stored at a low temperature of −18°C until used. The proximate analysis of this byproduct was conducted, including water, ash, fat, and protein content.

### 2.2. Gelatin Extraction

Before gelatin is extracted, pretreatment is completed with an acid and base solution. *Pangasius* skin was obtained according to the previous report by immersion of 100 g of the sample in 0.05 N NaOH solution for 1 hour with a sample ratio of 1 : 4 at room temperature [3]. The sample was then washed with water followed by immersion using 0.2% H_2_SO_4_ solution for 1 hour. Finally, the sample was soaked with 0.2% citric acid for 6 hours in the ratio of 1 : 4. The swim bladder sample was pretreated differently compared to the skin sample. 100 g sample was immersed in 0.05 N NaOH solution at room temperature with a sample ratio of 1 : 10 by weight for 1 hour. The sample was washed with distilled water to neutral pH followed by soaking using 0.2% citric acid solution for 1 hour while stirring with a sample ratio of 1 : 10 and then washed using distilled water until neutral. The pretreated samples of the skin, swim bladder, and mix of the skin and swim bladder (1 : 1 ratio) were extracted with deionized water at 65°C for 7 hours. The extracted samples were filtered, and then the filtrates were dried in an oven at 50°C for 24 hours to yield solid gelatin. The gelatin properties were characterized, including the ash content, pH, gel strength, viscosity, and protein profile analysis (SDS-PAGE).

### 2.3. Hard Capsule Casting

10 g gelatin powder is mixed with 10 mL distilled water at 85–90°C until all gelatin dissolved into homogenous solution. Following the reported method [10], the preheated pin bar is then dipped into the warm mixed solution and immediately removed when the film is formed and dried for approximately 30 minutes. Dried capsules were released from the pin bar, and then the properties of the capsules were analyzed including the weight and thickness, ash and moisture contents, and the time evaluation of capsule shell rupture.

## 3. Results and Discussion

### 3.1. Proximate Analysis of the *Pangasius* Skin and Swim Bladder

Proximate analysis is conducted to determine the quality of the starting material that will be used for the gelatin extraction. Water, ash, fat, and protein contents of raw material affect the properties of the manufactured gelatin significantly [11]. The proximate result of the *Pangasius* skin and swim bladder is shown in Table 1. The proximate composition of the skin and swim bladder produced is quite different compared with the previous report given by Mahmoodani et al. [12] and Vijayan et al. [13] for the proximate analysis of the *Pangasius* skin and swim bladder, respectively. These differences were due to different *Pangasius* species use. In the study of Mahmoodani et al. [12], *Pangasius sutchi* was used and originally from the waters of Penang, Malaysia, while the *Pangasius* species in the study of Vijayan et al. [13], namely, *Pangasius hypophthalmus*, was obtained from the waters of the western district of Godavari, Andhra Pradesh, India.

The *Pangasius* skin and swim bladder have ash content values of less than 0.5%. The value of the ash content less than 0.5% is a good-quality raw material for the manufacture of collagen and gelatin [9]. However, the amount of fat in the skin material is relatively high compared to that in the swim bladder, 12.3% and 0.25%, respectively. Thus, to improve the quality of the resulting gelatin, the optimization of the pretreatment process is required [14]. Because of the disparity in tissue composition where the *Pangasius* skin has a hypodermic layer or a subcutaneous layer in which it is composed of fat tissue, the fat content in *Pangasius* skin is higher than in the *Pangasius* swim bladder, where that layer is not stored as swimming bubbles. The protein content of the *Pangasius* skin and swim bladder is 39.8% and 27.6%, respectively, and it is in good agreement with the previous report. However, this value is relatively low compared to the protein content of the *Pangasius* bone which is 40.9%.

### 3.2. Physicochemical Properties of Extracted Gelatin

The physicochemical properties of the *Pangasius* skin and swim bladder are shown in Table 2. Gelatin from the *Pangasius* skin and swim bladder was successfully extracted with the yield in the range of 19–23% (Table 2). The yield of gelatin can be influenced by several factors, including the type of material, the pretreatment process, and the extraction temperature [15]. The gelatin yield in this study was higher than in other previous reports of gelatin extraction from other fish resources. Gelatin yields 11.3% from tuna skin [15]. Gelatin yield of yellowfin tuna skin is 19.97% [3]. Gelatin extraction of the goldfish swim bladder had yielded 13.5% by pretreatment using NaOH, sulfuric acid, citric acid, and extraction temperatures of 45–50°C [9].

pH is an essential factor for the quality of gelatin. The results of pH values based on Table 2 show that swim bladder gelatin has the highest pH value of 5.16, whereas skin gelatin has the lowest pH value of 4.15. The pH value of gelatin is in accord with the standard of the Gelatin Manufacturer's Institute of America for edible gelatin which states that the pH value of gelatin is around 3.8–5.5 [16].

Gel strength is the most important physical property of gelatin. The strength of the gel illustrates the cohesion power between gelatin molecules and is proportional to the molecular weight. The results of gelatin gel strength values based on Table 2 show that mixed gelatin has the highest gel strength of 278 bloom, whereas skin gelatin has the lowest gel strength of 238 bloom. The results are in accord to the standard of the Gelatin Manufacturer's Institute of America for edible gelatin which states that the strength of gelatin gel is around 50–300 bloom [16].

The gel strength of this gelatin is comparable with gelatin from bovine [17] and other fish sources [18–21]. It is widely reported that gelatin from tropical fish species displays higher gel strength compared to gelatin from cold water fish varieties. The high bloom value of *Pangasius* waste gelatin found in the current investigation exhibits its suitability for the application as hard capsule shells. Beside gel strength, viscosity is also the essential property to assess the gelatin quality. The viscosity of extracted gelatin is shown in Table 2. Gelatin from the *Pangasius* swim bladder has the highest viscosity of 75 mP, while the skin and mixed gelatin showed the viscosity value of 61 and 71 mP, respectively. These viscosity values were proportional to bovine and porcine gelatin and thus showed the potential of *Pangasius* waste gelatin for commercial purposes.

### 3.3. SDS-PAGE Profile of Gelatin from *Pangasius* Wastes

**Figure 1:**
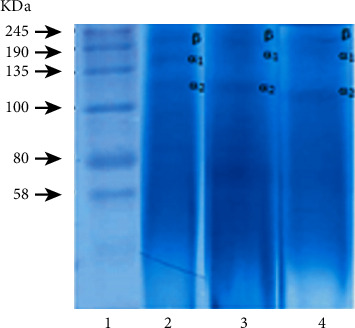
(1) SDS-PAGE profile of gelatin from *Pangasius* waste: molecular weight marker and (2) gelatin from the skin, (3) swim bladder, and (4) mixed.

Protein profile is one of the critical properties for the quality of extracted gelatin. The relative amount of the protein component, including *α*- and *ß*-protein fragments, has a significant effect on the physical properties of gelatin. In Figure 1, the SDS-PAGE analysis showed the protein profile of *Pangasius* waste gelatin. The *Pangasius* skin, swim bladder, and mixed gelatin showed a similar protein pattern with three major bands around 128–230 kDa that were corresponding to *α*1-, *α*2-, and *β*-protein components akin to the previous reports. The emergence of *α*- and *β*-chains and other lower molecular weight protein fragments occurs during extraction due to the collagen degradation process. Gelatin of yellowfin tuna skin extracted at a temperature of 65°C had a molecular weight of *β* 250 kDa, *α*1 129.670 kDa, and *α*2 116.364 kDa [19]. The *β*-component shows high molecular weight (>200 kDa), while the *α*1- and *α*2-components show low molecular weight (>120 kDa) [22].

### 3.4. Hard Capsule Shells

**Figure 2:**
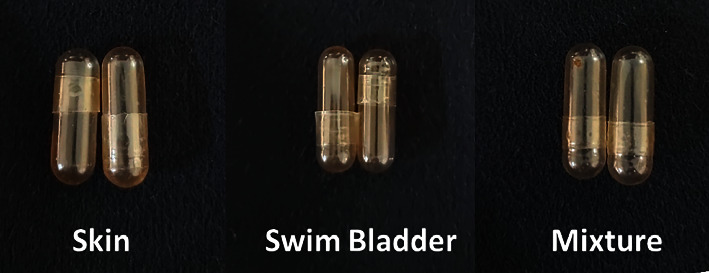
Hard capsule shells from gelatin of *Pangasius* waste.

In the current study, gelatin from *Pangasius* waste was used as material for hard capsule shells.  Figure 2 shows the image of capsules produced from the *pangasius* skin, swim bladder, and mixed gelatin. The transparency of the capsules was respectable and similar regardless of the gelatin origin, although the color of the capsules is slightly yellow. Physical properties of hard capsule shells from *pangasius* skin and swim bladder gelatin are shown in Table 3, and the properties were as good as the commercial mammalian-based gelatin capsules thus showing the feasibility of the use of gelatin from *Pangasius* wastes as the base material to produce the hard capsule shells.

The results of the characteristics of skin, swim bladder, and mixed gelatin capsules can be seen in Table 3. The produced capsule dimensions from three types of gelatin are different substantially (*p* < 0.05) due to the variance of dipping temperature and viscosity of the gelatin sources, while the influence on the filling of capsule base material also contributes to the difference in capsule thickness.

**Figure 3:**
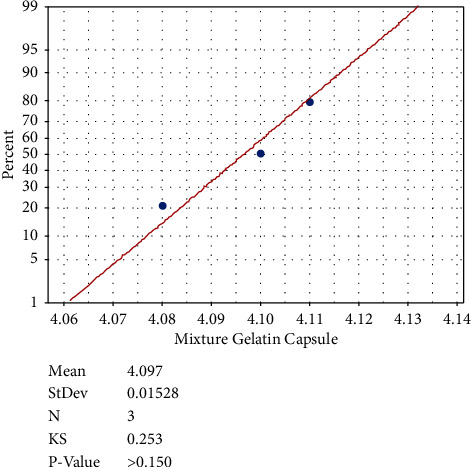
Kolmogorov–Smirnov plot of disintegration time of the mixed gelatin capsule.

The size of the capsules based on the dimensions of the capsules produced in this study is included in capsule size 0 [23]. Capsules have various variations and sizes. It is intended to adjust the capsule shell with medicinal ingredients to be inserted into the capsule, such as powder, paste, and liquid [7]. The values of capsule weight typically spread (*p* > 0.05) based on Kolmogorov–Smirnov analysis, which indicates the influence of the raw gelatin material on the weight of the capsules produced (*p* < 0.05). The water content of capsules is influenced by temperature and drying time of the dipping process, as well as the physical properties and the viscosity of gelatin [24]. The capsule disintegration time is normally spread (*p* > 0.05) based on Kolmogorov–Smirnov analysis (Figure 3). Furthermore, the results of Duncan's test showed that the disintegration time of the skin gelatin capsule differs significantly with the swim bladder gelatin capsule and mixed gelatin capsule (Table 4).

## 4. Conclusions

The extraction of gelatin from the *Pangasius* skin, swim bladder, and mixture has been effectively performed with the yields in the range of 19 to 21%. The physicochemical properties of extracted gelatins are comparable to the mammalian-based gelatins with the values of gel strength being 239, 273, and 278 bloom for *Pangasius* skin, swim bladder, and mixture, respectively. The physical properties of hard capsule shells from these gelatins were comparable to the standards of commercial capsules.

## Figures and Tables

**Table 1:** Proximate analysis of *Pangasius* waste materials.

Parameter (% w/w)	Skin		Swim bladder	
	This work	*Pangasius sutchi* [10]	This work	*Pangasius hypophthalmus* [11]
Moisture	39.08 ± 0.65	50.03 ± 0.27	71.24 ± 0.20	73.9 ± 0.50
Ash	0.26 ± 0.020	4.14 ± 0.18	0.10 ± 0.01	0.89 ± 0.10
Fat	12.33 ± 0.23	6.95 ± 0.17	0.25 ± 0.01	3.77 ± 0.10
Protein	39.75 ± 0.12	30.91 ± 0.28	27.56 ± 0.37	20.50 ± 0.20

**Table 2:** Physicochemical properties of gelatin.

Properties	Gelatin			
	Skin	Swim bladder	Mixture	Standard [21]
Yield (%)	19.09 ± 0.34	19.92 ± 0.53	23.51 ± 0.93	—
Moisture (%)	9.20 ± 0.020	7.61 ± 0.27	8.58 ± 0.19	Max 15
Ash (%)	1.10 ± 0.20	1.77 ± 0.81	1.10 ± 0.53	Max 5
Acidity (pH)	4.15 ± 0.01	5.16 ± 0.01	5.03 ± 0.03	3.8–5.5
Gel strength (bloom)	238.88 ± 1.43	272.85 ± 1.45	278.50 ± 4.89	50–300
Viscosity (mP)	65 ± 0.36	74.7 ± 0.06	71 ± 0.20	15–75

**Table 3:** Physical properties of hard capsule shells from *Pangasius* skin and swim bladder gelatin.

Parameter	Hard capsule shells			Commercial capsule [9]
	Skin	Swim bladder	Mixture	
Body length (mm)	19.22 ± 0.06^c^	18.26 ± 0.01^b^	18.04 ± 0.18^a^	18.66 ± 0.30
Body diameter (mm)	7.21 ± 0.03^a^	7.36 ± 0.03^b^	7.32 ± 0.05^b^	7.36 ± 0.02
Caps length (mm)	11.28 ± 0.02^b^	10.79 ± 0.28^a^	10.63 ± 0.12^a^	10.98 ± 0.36^b^
Caps diameter (mm)	7.52 ± 0.06^a^	7.49 ± 0.02^a^	7.70 ± 0.09^b^	7.67 ± 0.04
Weight (mg)	104 ± 2.65^b^	90.33 ± 4.16^a^	112.67 ± 2.52^c^	—
Acidity (pH)	4.40 ± 0.03^a^	5.14 ± 0.04^b^	5.13 ± 0.04^b^	5.89 ± 0.049
Moisture (%)	13.04 ± 0.03^a^	14.17 ± 0.06^c^	13.19 ± 0.07^b^	14.12 ± 0.262
Rupture times (min)	5.05 ± 0.05^b^	4.05 ± 0.05^a^	4.10 ± 0.02^a^	7.39 ± 0.113

The values represent the mean and standard deviation. Significant differences at P<0.05 are indicated by different letters in a column (a, b, c).

**Table 4:** Duncan's test of disintegration time of hard capsule shells.

Hard capsule shells	*N*	Subset for alpha = 0.05	
		1	2
Swim bladder	3	4.0500	
Mixed	3	4.0967	
Skin	3		5.0500
Sig.		0.220	1.000

## Data Availability

The data used to support the findings of this study are available from the corresponding author upon request.
